# Quinacrine Induces Nucleolar Stress in Treatment-Refractory Ovarian Cancer Cell Lines

**DOI:** 10.3390/cancers13184645

**Published:** 2021-09-16

**Authors:** Derek B. Oien, Upasana Ray, Christopher L. Pathoulas, Ling Jin, Prabhu Thirusangu, Deokbeom Jung, Joseph E. Kumka, Yinan Xiao, Sayantani Sarkar Bhattacharya, Dennis Montoya, Jeremy Chien, Viji Shridhar

**Affiliations:** 1 Division of Experimental Pathology and Laboratory Medicine, Mayo Clinic, 200 First Street SW, Rochester, MN 55905, USA; derek.oien@astrazeneca.com (D.B.O.); ray.upasana@mayo.edu (U.R.); pathoulas@uchc.edu (C.L.P.); jin.ling1@mayo.edu (L.J.); Thirusangu.Prabhu@mayo.edu (P.T.); jungdb@hotmail.com (D.J.); Joseph.kumka@gmail.com (J.E.K.); yinanxiao1203@gmail.com (Y.X.); bhattacharya.sayantani@mayo.edu (S.S.B.); 2 University of Connecticut Health Center-Medical School, Farmington, CT 06032, USA; 3 ASAN Biomedical Research Center, Seoul 138-736, Korea; 4 University of Minnesota Medical School, Minneapolis, MN 55455, USA; 5 Department of Obstetrics and Gynecology, The Second Xiangya Hospital, Central South University, Changsha 410008, China; 6 Department of Biochemistry and Molecular Medicine, University of California Davis Health, 2700 Stockton Boulevard, Sacramento, CA 95817, USA; djmontoya@ucdavis.edu (D.M.); jrchien@ucdavis.edu (J.C.)

**Keywords:** quinacrine, ribosomal biogenesis, nucleolar stress, nucleostemin, chemotherapy resistance, ovarian cancer

## Abstract

**Simple Summary:**

A high mortality rate in ovarian cancer imposes the need for improved therapy; most patients initially respond to systemic chemotherapy but later relapse with fatal treatment-refractory tumors. We previously evaluated repurposing the antimalarial agent quinacrine as an anticancer agent in chemoresistant ovarian cancer. Quinacrine has a long history of use in humans and we have demonstrated selectivity characteristics for reducing drug resistant tumors. In this study, we analyze different responses to quinacrine between drug resistant and sensitive cell lines to identify major pathways related to this selectivity. We confirm our results in notoriously drug resistant high-grade serous ovarian cancer cells and describe nucleostemin as a new quinacrine target related to ribosomal biogenesis and nucleolar stress. This study provides preclinical evidence that quinacrine may be effective against relapsed/refractory ovarian cancer.

**Abstract:**

A considerable subset of gynecologic cancer patients experience disease recurrence or acquired resistance, which contributes to high mortality rates in ovarian cancer (OC). Our prior studies showed that quinacrine (QC), an antimalarial drug, enhanced chemotherapy sensitivity in treatment-refractory OC cells, including artificially generated chemoresistant and high-grade serous OC cells. In this study, we investigated QC-induced transcriptomic changes to uncover its cytotoxic mechanisms of action. Isogenic pairs of OC cells generated to be chemoresistant and their chemosensitive counterparts were treated with QC followed by RNA-seq analysis. Validation of selected expression results and database comparison analyses indicated the ribosomal biogenesis (RBG) pathway is inhibited by QC. RBG is commonly upregulated in cancer cells and is emerging as a drug target. We found that QC attenuates the in vitro and in vivo expression of nucleostemin (NS/GNL3), a nucleolar RBG and DNA repair protein, and the RPA194 catalytic subunit of Pol I that results in RBG inhibition and nucleolar stress. QC promotes the redistribution of fibrillarin in the form of extranuclear foci and nucleolar caps, an indicator of nucleolar stress conditions. In addition, we found that QC-induced downregulation of NS disrupted homologous recombination repair both by reducing NS protein levels and PARylation resulting in reduced RAD51 recruitment to DNA damage. Our data suggest that QC inhibits RBG and this inhibition promotes DNA damage by directly downregulating the NS–RAD51 interaction. Additionally, QC showed strong synergy with PARP inhibitors in OC cells. Overall, we found that QC downregulates the RBG pathway, induces nucleolar stress, supports the increase of DNA damage, and sensitizes cells to PARP inhibition, which supports new therapeutic stratagems for treatment-refractory OC. Our work offers support for targeting RBG in OC and determines NS to be a novel target for QC.

## 1. Introduction

Deregulated ribosome biogenesis (RBG) and nucleolar hypertrophy are established hallmark features of neoplastic cells [1]. Increased RBG supports rapid cell proliferation as the proliferation rate in tumors is directly proportional to nucleolar size and RNA polymerase (Pol I) activity [2]. Pol I exclusively transcribe ribosomal RNA (rRNA) from ribosomal DNA (rDNA) genes within the nucleolus and is responsible for forming the 47S rRNA precursor, which is later processed into the 18S, 5.8S and 28S mature rRNAs [1,3]. rRNA transcription is the rate-limiting step in RBG and has been shown to be regulated by many different factors [4,5]. The loss of tumor suppressors such as p53 or pRb can stimulate RBG and promote rapid cell proliferation [6,7,8,9,10,11]. Given that RBG is commonly upregulated in cancer cells compared to non-neoplastic cells, there is a therapeutic window for exploiting its inhibition as a therapeutic strategy against cancer progression. Pol I inhibitors modulate RBG and can induce nuclear stress [12] as well as DNA damage [13]. Selective RBG inhibitors, including CX-54613 [14,15,16,17,18], have been developed as anticancer agents with limited effects on normal cells. CX-5461 prevents the Pol I transcription initiation factor SL-1 from binding to the rDNA promoter and is currently being evaluated in a Phase I clinical trial for breast cancer (NCT02719977). Disruption of RBG promotes nucleolar stress that is accompanied by changes in nucleolar structure and protein localization including the formation of nucleolar caps consisting of nucleolar proteins such as upstream binding factor (UBF) and fibrillarin (FBL) [19,20]. Nucleolar stress induced by RBG inhibitors can release ribosomal proteins from the nucleoplasm where they bind and inhibit MDM2, which promotes p53 activation and triggers apoptosis [21,22]. In addition, nucleolar proteins including poly(ADP-ribose) polymerase-1 (PARP1) and nucleostemin (NS) have been associated with the DNA damage response [23]. NS is a stem cell-enriched protein that recruits the homologous recombination RAD51 protein to double-strand DNA breaks [24,25]. Therefore, targeting Pol I transcription and activating p53 in cancer cells has developed into a promising anticancer approach [26].

Quinacrine (QC) is an antimalarial drug that has potential anticancer indications with limited toxicity towards non-malignant cells [27]. Several QC anticancer mechanisms have been described for a variety of different cancer types [28,29,30]. We previously reported that QC has higher efficacy in chemotherapy-resistant gynecologic cancer cells [31]. In this study, we aimed to identify QC cytotoxic mechanisms related to drug resistance by comparing the transcriptome changes to isogenic sensitive cell lines. Expression analyses indicated RBG was inhibited in artificially generated drug resistant cells, which we confirmed using high-grade serous ovarian cancer (OC) cells with intrinsic drug resistant phenotypes. QC has been shown to inhibit RBG in leukemia cell lines [32,33,34], but this has not been demonstrated in solid tumors. Our results suggest QC-induced RBG inhibition promotes nucleolar stress conditions and cell death in treatment-refractory OC cells. Based on recent PARP inhibitor approvals for OC and our data suggesting QC interrupts the DNA damage response, we also evaluated the combination of QC with PARP inhibitors.

## 2. Materials and Methods

### 2.1. Cell Culture and Reagents

Human ovarian cancer cell lines OVCAR5, OVCAR7, OVCAR 8 from ATCC (Manassas, VA, USA), HeyA8, HeyA8MDR, SKOV3TR cells from MD Anderson Cancer Center (Houston, TX, USA), PEO 1/4 cells from Fred Hutchinson Cancer Research Center, (Philadelphia, PA, USA), OV202 and OVCAR-8-DR-GFP cells from Mayo Clinic (Rochester, MN, USA), C13 and OV2008 from Ottawa Hospital Research Institute (Ottawa, ON, Canada) were grown as mentioned (Table S1) and the reagents used (Table S2). Isogenic pairs of ovarian cancer cell lines OV2008 (chemosensitive) and C13 (cisplatin resistant) cells derived from OV2008 [35]; HEYA8 (chemosensitive) and HEYA8MDR (carboplatin-resistant) and isogenic taxol-sensitive SKOV3 and taxol-resistant SKOV3TR cells [36] were used for the study.

### 2.2. RNA Isolation and Sequencing

Cells were treated with 8 µM QC or vehicle for 6 h [31]. RNA was isolated using TRIzol/chloroform protocol as described previously [37]. RNA libraries were prepared with TruSeq RNA library kit (Illumina, San Diego, CA, USA). Illumina transcriptome sequencing was performed at the Mayo Sequencing Core Facility. At least 20 million mapped reads were analyzed for each RNA library.

### 2.3. Transcript Analyses

TopHat [38] was used to align reads to the Human Reference Genome (hg19), and HTSeq [39] was used to produce read counts. A heatmap was generated with Morpheus software (Broad Institute, Cambridge, MA, USA). Differential gene analysis was performed with BRB-ArrayTools [40], DESeq2 [41], and Ballgown pairwise comparison to identify differentially expressed genes with a *p*-value of <0.05. The list of genes generated was evaluated by Metascape Ingenuity Pathway Analysis and Panther Analysis [42]. Hypergeometric *p*-values and enrichment factors were used for filtering to generate a hierarchically clustered tree based on Kappa-statistical (0.3 score) similarities among gene memberships. The 617 genes induced by QC that differed between the sensitive and resistant cells was analyzed in the Enrichr database (WikiPathways 2021, KEGG 2021, and Jensen Compartments). https://maayanlab.cloud/Enrichr/enrich?dataset=b434bfb5b433dbb60c6edf0b2358f8a4 (accessed on 4 July 2021).

### 2.4. Quantitative RT-PCR

Expressions of selected genes were validated by SYBR-Green qPCR as previously described [43] with primers synthesized by Integrated DNA Technologies (Coralville, IA, USA) listed in Table S3. Reaction was executed using a CFX96 Real-Time PCR system (Bio-Rad, Hercules, CA, USA) with normalization to RPLP0 expression.

### 2.5. Western Blots

Whole cell lysates were subjected to immunoblot analysis [44] with primary antibodies listed in Table S2. For puromycin labeling, 10 µM puromycin was added for the last hour of treatment as described previously [45]. Protein bands were visualized by fluorophore-conjugated secondary antibodies (LI-COR) and imaged using LI-COR Odyssey Fc Imaging System (Lincoln, NE, USA).

### 2.6. Generation of Knockdown Stable Clones

OVCAR5 cells were stably knocked down for NS using targeted shRNA (Sequence: CCTTGGACAAACAGATCACAA) and for RPA194 (sh1_Sequence: CCGGGACGAGATGAATGCCCATTTCCTCGAGGAAATGGGCATTCATCTCGTCTTTTTG, sh2_Sequence: CCGGGCCAACTGCAAGGCCTATAATCTCGAGATTATAGGCCTTGCAGTTGGCTTTTTG) from Sigma Aldrich and following the manufacturer protocol.

### 2.7. Immunofluorescence Staining

To visualize 5-fluorouridine labeling and fibrillarin protein by immunofluorescence, cells plated on chamber slides were treated with QC. 5-fluorouridine (2 mM) was added for 10 min. Cells were fixed, permeabilized and probed with either BrdU or fibrillarin antibody for 2 h followed by fluorescent-conjugated secondary antibody. Nuclei were stained with DAPI. Slides were imaged on an Evos (Life Technologies, Carlsbad, CA, USA) microscope.

### 2.8. Clonogenic Assay

OVCAR5 NTC and sh_NS knockdown cells were seeded at 500 cells/well in 6-well plates, incubated for 9 days and visualized with crystal violet stain. Colonies were quantified as described previously [44].

### 2.9. Synergy Assay

Approximately 500 cells/well were plated onto 6-well plates and treated with QC, rucaparib, or a combination of both continuously for up to 14 days until colonies became visible and stained. The colonies were counted using ImageJ software. To determine synergy, combination index (CI) values were calculated from a range of drug concentrations by CompuSyn software using a non-constant ratio approach according to Chou-Talalay. The CI values were calculated; CI < 1 indicates a synergistic effect, CI < 0.7 indicates a significant synergistic effect [46]. The values represent the mean ± SD of three independent experiments.

### 2.10. Homologous Recombination (HR) Assay

OVCAR-8 cells stably transfected with pDR-GFP, an HR substrate that creates a functional GFP upon successful HR by I-SceI cleavage, was obtained [47]. For studies with shRNAs, OVCAR-8-DR-GFP cells were transfected twice, first electroporated with the NS shRNA. On day 2, the cells were electroporated with NS shRNA plus ipCβASceI plasmid (encoding I-SceI) and analyzed for GFP fluorescence on day 5 by flow cytometry.

### 2.11. HeyA8-MDR Xenografts

We previously reported the QC efficacy study for HeyA8-MDR xenografts, which followed a protocol approved by the Mayo Foundation IACUC [31]. Here, we performed immunoblot analysis with protein lysates from these xenografts.

### 2.12. Statistical Analysis

All studies were performed for 3 independent experiments in triplicates unless mentioned. Data were expressed as mean ± standard deviation. Significant changes (* *p* < 0.05) were determined using student’s *t*-test unless otherwise noted.

## 3. Results

### 3.1. QC Treatment Modifies the RNA Expression Changes in Chemosensitive and Chemoresistant Cells

**Figure 1:**
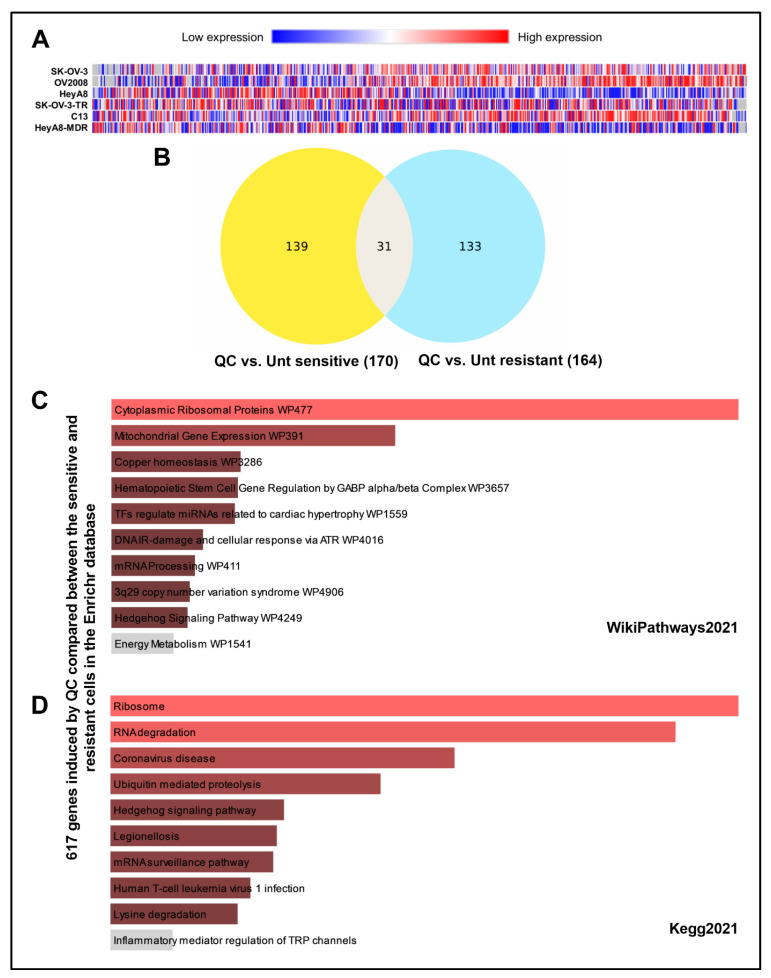
QC-induced differential gene expression in chemotherapy sensitive and resistant isogenic cells. (**A**) Heatmap of 616 gene transcripts identified in secondary analysis of resistant cells to have significant (*p* < 0.05) differential gene expression when treated with QC. Shades of blue represent decreases in abundance and shades of red represent increases in abundance. Genes (horizontal axis) are ordered from highest to lowest fold increase in average fold change comparing resistant to sensitive cells treated with QC. (**B**) Venn diagram of differentially expressed genes from quinacrine treatment of sensitive (left) and resistant (right) cells, and genes common to both sets (middle). (**C**) QC-induced 617 genes that differed between the sensitive and resistant cells were analyzed in the Enrichr database and the representative pathway was shown in WikiPathways 2021 and (**D**) KEGG 2021 analysis. Abbreviations: QC: Quinacrine; Unt: Untreated.

The RNA sequencing secondary analysis revealed 616 transcripts that were significantly (*p* < 0.05) differentially expressed in SK-OV-3-TR, C13, and HeyA8-MDR resistant cell lines when treated with QC (Figure 1A). Additional filtering analyses identified 170 differentially expressed genes in sensitive cells, 164 differentially expressed genes in resistant cells, and 31 transcripts common to both sets upon QC treatment (Figure 1B).

The QC-induced differentially expressed gene sets were submitted to the Metascape database35 and database conversion resulted in 117 and 120 Human Entrez Gene IDs from resistant and sensitive cell lines, respectively. After filtering statistically enriched terms, the analysis identified major signaling and hallmark pathways correlated to QC-induced transcription profiles for resistant cells (Figure S1A) and sensitive cells (Figure S1B). The hallmark p53 pathway was correlated to both resistant and sensitive cell line gene sets (Figure S1A,B), which has previously been reported to be altered by QC in cancer cells [27]. Several signaling pathways of nucleotide and DNA regulation (protein-DNA complex assembly, modification, metabolic process, and replication) were perturbed, mainly in resistant cells (Figure S1A), suggesting nucleotide regulation pathways may be important for QC mechanisms in drug resistant cancer cells. Furthermore, the 617 QC-induced genes that varied between the sensitive and resistant cells were subjected to analysis in the Enrichr database. This shows more RNA/ribosome pathway alteration in the WikiPathways 2021, KEGG 2021, and Jensen Compartments analysis (Figure 1C,D and Figure S1C).

Gene expression analysis from RNA-seq showed upregulation of Ras GTPase-activating protein-binding protein 1 (G3BP1), a marker for stress granules in both sensitive and resistant OC cells upon QC treatment (Figure S2A,B). Candidate genes were selected for validation based on a causal relationship to pathway correlation analysis, high-fold change, and most significant *p*-values for RNA sequencing data after QC dosing. Selected transcripts included GNL3/NS, ASNS, DNMT3B, CSTM, BOP1, and PHDGDH in OC resistant cells (Figure S2C–H). qPCR analysis revealed that GNL3/NS, which plays a role in pre-rRNA processing, was significantly downregulated by QC treatment in the OC resistant cell types (Figure S2C). Similarly, ASNS, the gene for asparagine synthetase that regulates serine metabolism and nucleotide synthesis [48], was downregulated after QC treatment (Figure S2D). CST6 (CSTM), a lysosomal cysteine protease inhibitor was induced by QC in the resistant cells by 5.4-fold and expression of DNMT3B was downregulated by QC (Figure S2E,F). DNMT3B expression and activity can contribute to CSTM promoter methylation [49] and promote chemoresistance [50,51]. QC treatment increased the RBG-associated [52] BOP1 in the resistant cells (Figure S2G) and decreased PHGDH (Figure S2H). Western analysis in HeyA8-MDR, OVCAR8, and OVCAR5 cells also showed a similar reduction in the expression levels of DNMT3B, PHGDH, and ASNS, while CSTM was upregulated upon QC treatment for 24 h (Figure S1I–K). Together, these results suggest that QC treatment alters the nucleotide regulation and the ribosomal pathways in the OC cells.

### 3.2. QC Inhibits Ribosome Biogenesis and Induces Nucleolar Stress in OC

#### 3.2.1. QC Treatment Attenuates RBG in High-Grade Serous OC Cells

**Figure 2:**
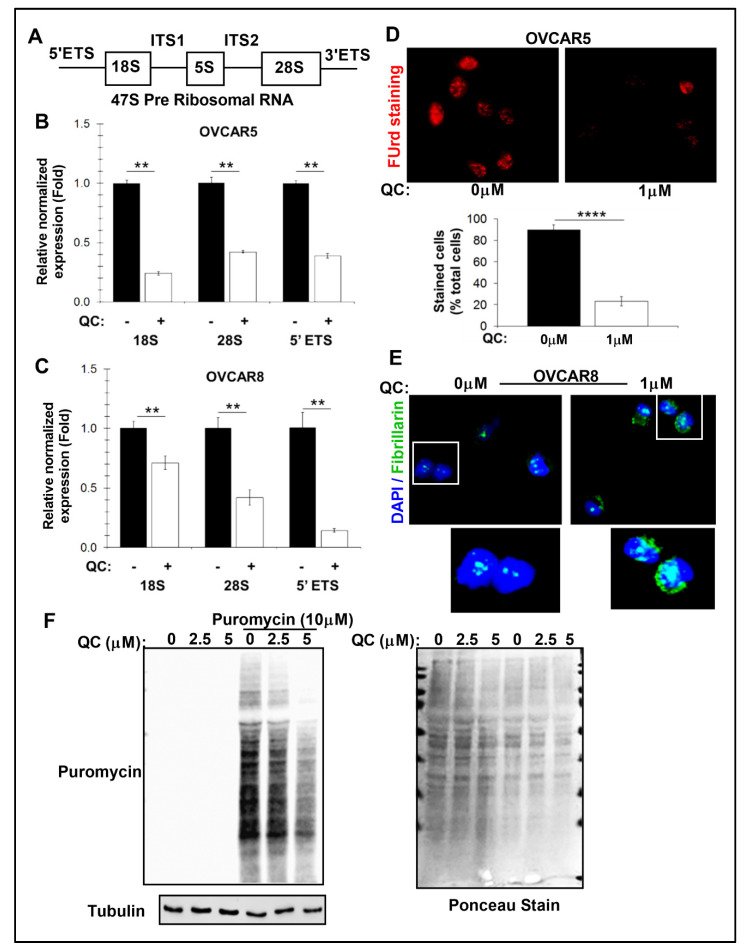
QC impairs RBG and induces nucleolar stress in OC cells. (**A**) Graphic representation of 47S pre-ribosomal RNA. ETS–external transcribed spacers, ITS–internal transcribed spacers. (**B**) Relative expression changes for ribosomal subunits after 1 h of 1 µM QC treatment in OVCAR5 and (**C**) OVCAR8 cells (normalized to RPLP0; *n* = 3). Error bars represent standard error, and significance is denoted by ** (*p* < 0.01). (**D**) Incorporation of 5-fluorouridine was monitored after 10 min of labeling in OVCAR5 cells treated with 1 µM QC for 2 h. Representative images were shown, quantified and shown as bar graph **** (*p* < 0.0001). (**E**) Fibrillarin (Green) IF staining was performed after 2 h of 1 µM QC in OVCAR8 cells. DAPI was used to stain nucleus. (**F**) Whole cell lysates of OVCAR5 were analyzed for puromycin incorporation (1 h, 10 µM) with increasing QC concentrations. Membrane with Ponceau stain was shown for protein loading. Abbreviations: QC: Quinacrine; ITS1/2: internal transcribed spacers 1 and 2; 5′/3′ ETS: external transcribed spacers; FUrd: 5-fluorouridine.

The QC-induced attenuation of RBG was confirmed in high-grade serous OC cells. In RBG, the 18S, 5.8S, and 28S rRNAs are transcribed by RNA polymerase I from a single transcription unit of 47S pre-ribosomal RNA in the nucleolus (Figure 2A). The three rRNAs are interspersed with non-coding sequences, specified by 5′ and 3′ external transcribed spacers (5′ ETS and 3′ ETS) and internal transcribed spacers 1 and 2 (ITS1 and ITS2). Quantitative RT-PCR showed repression of 47S rRNA as assessed by the levels of short-lived 5′ ETS, 18S, and 28S after the addition of QC in OVCAR5 and OVCAR8 cells (Figure 2B,C). This repression also correlated to a decrease in 5-fluorouridine incorporation (FUrd), suggesting a decrease in newly synthesized RNA in OVCAR5 and OVCAR8 cells (Figure 2D and Figure S3A). QC promotes FBL redistribution in the form of extranuclear foci and nucleolar caps of this pre-ribosomal RNA methyltransferase in both the cells (Figure 2E and Figure S3B), which is an indicator of nucleolar stress conditions [45]. These QC-induced changes correlated to reduced puromycin labeling for protein translation in OVCAR5 cells (Figure 2F). Overall, these results suggest QC attenuates the biosynthesis and processing of rRNA, which may be inducing a nucleolar stress environment.

#### 3.2.2. QC-Induced Ribosomal Stress Downregulates NS and RPA194

**Figure 3:**
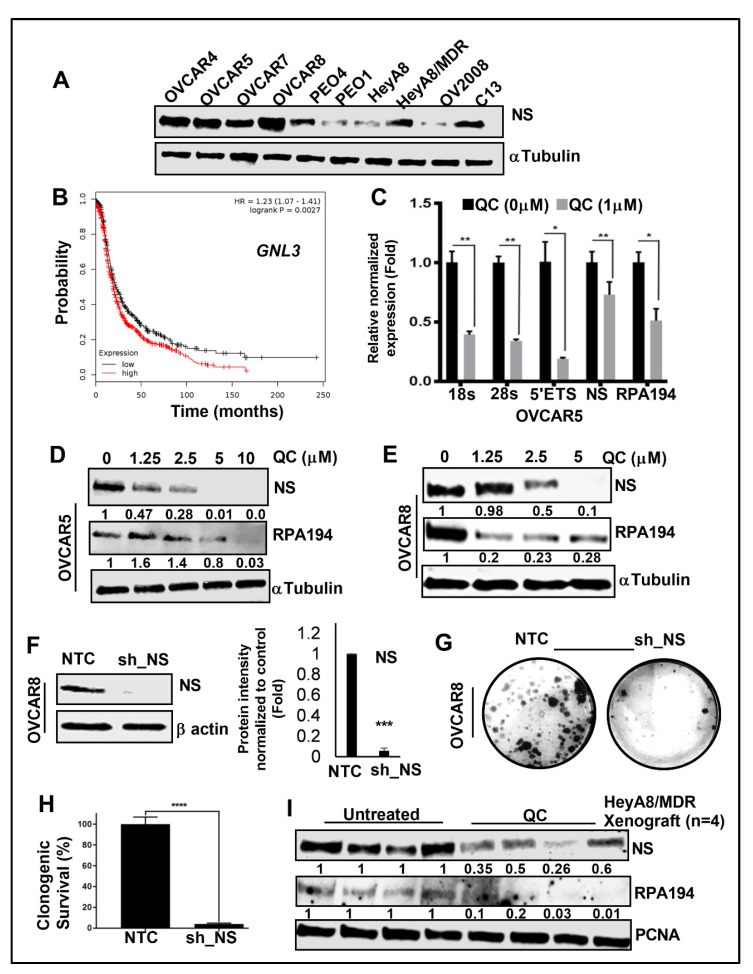
Nucleolar protein NS and RPA194 is downregulated by QC treatment. (**A**) NS protein expression in various OC cell lines was assessed by western blot. αTubulin was used as loading control. (**B**) Kaplan–Meier progression-free survival (PFS) analysis shows high GNL3/NS expression is associated with worse PFS in OC patient’s cohort. (**C**) Relative expression changes for ribosomal subunits NS and RPA194 after 1 h of 1 µM QC treatment in OVCAR5 OC cells (normalized to RPLP0; *n* = 3) * (*p* < 0.05), ** (*p* < 0.01). (**D**) Immunoblot analysis of NS and RPA194 expression in OVCAR5 and (**E**) OVCAR8 cells upon QC treatment for 24 h in a dose dependent manner. α Tubulin was used as loading control. Densitometric analysis was performed using Image J, normalized to endogenous control and the fold change was provided beneath the panel. (**F**) shRNA-mediated stable NS knockdown OVCAR8 cells were generated, and the knockdown effect was validated by western blot analysis. αTubulin was used as loading control. Densitometric analysis was performed using Image J, normalized and fold change was plotted *** (*p* < 0.001). (**G**,**H**) Clonogenic OVCAR8 cell viability after NS knockdown compared to NTC transfection (*n* = 3). Error bars represent standard error, and significance is denoted by **** (*p* < 0.0001). (**I**) Western blot analysis was performed in untreated control and QC-treated HeyA8-MDR OC xenograft tumors against NS and RPA194 expressions. PCNA was used as loading control. Fold change was analyzed and provided beneath the panel. Abbreviations: QC: Quinacrine; NS/GLN3: Nucleostemin, RPA194: RNA Polymerase I Subunit A; NTC: non-targeted control, sh_NS: shRNA-mediated NS downregulation; PCNA: Proliferating cell nuclear antigen.

QC-induced ribosomal stress downregulates NS and RPA194. NS was initially reported to regulate p53 signaling and later found to also regulate pre-ribosomal rRNA processing [53]. NS is expressed in several OC cell lines with higher expression in HeyA8/MDR and C13 compared to parental HeyA8 and OV2008 cells, respectively (Figure 3A), and higher expression of NS was significantly associated with lower progression-free survival for OC patients (Figure 3B). Dosing OVCAR5 and OVCAR8 cells with QC downregulates NS and RPA194 mRNA expression along with the RBG genes (Figure 3C and Figure S4A). Similar downregulation was obtained at the protein level in OVCAR5 cells (Figure 3D and Figure S4B,C), but did not decrease the nucleolar protein UBF (Figure S4D). OVCAR8 cells also showed downregulation of both NS and RPA194 (Figure 3E and Figure S4E,F) upon QC treatment. Likewise, high expression of RPA194 was also found to be associated with poor prognosis in OC patients (Figure S4G). Interestingly, QC has been shown to bind to G-quadraplexes in DNA, which is a characteristic of other ribosomal biogenesis inhibitors such as CX-5461 suggesting that QC could also disrupt Pol I transcription by a similar mechanism [18]. For comparison, OVCAR8 cells were dosed with CX-5461, which also resulted in inhibition of the 5′ETS target gene (Figure S4H) and reduced NS and RPA194 protein (Figure S4I). Stable NS knockdown by shRNA in OVCAR cells (Figure 3F) severely inhibited colony forming abilities (Figure 3G,H). Our prior studies demonstrated that treatment with QC in HeyA8-MDR xenograft mice reduces tumor weight [31], and here we found the expression of NS and RPA194 is also decreased in these tumor samples (Figure 3I and Figure S5A,B). These results suggest QC downregulates NS and RPA194 and induces nucleolar stress, which curtails growth in drug-refractory OC cells.

### 3.3. QC Treatment and NS Knock Down Disrupts HR Repair in OC Cells

**Figure 4:**
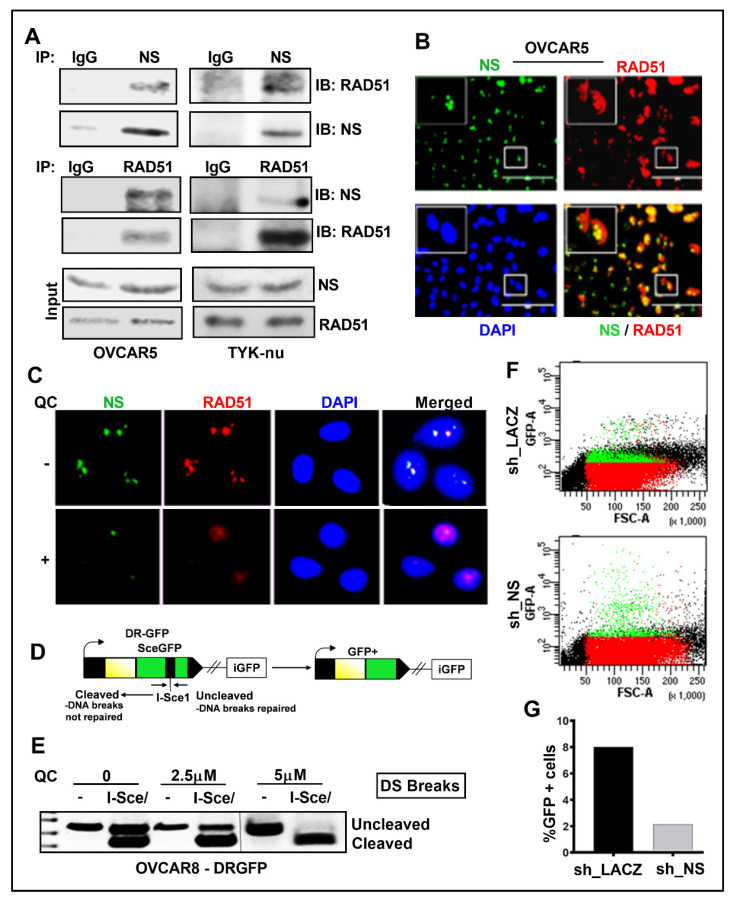
QC impairs the association of NS with RAD51 in OC cells. (**A**) OVCAR5 and TYK-nu cell extracts were immunoprecipitated with anti-NS and the co-precipitated RAD51 was detected by western analysis and vice versa. (**B**) IF analysis shows NS (Green) colocalizes with RAD51 (Red) (Merged image-yellow) in OVCAR5 cells. DAPI was used to stain the nuclei. (**C**) IF analysis shows that treatment with 2.5 µM QC disrupts NS (Green)–RAD51 (Red) association. DAPI was used to stain the nuclei. (**D**) PCR strategy for evaluating repair by HR. (**E**) Expression of I-SceI in OVCAR-8DR-GFP cells was performed to assess the repair of the DSB in the cells. Cells that can undergo HR repair through a short-tract gene conversion without crossing over become GFP positive. Representative gel image was shown after PCR amplification and digestion with I-SceI. (**F**,**G**) Repair is measured as the percentage of GFP positive cells in OVCAR-8DR-GFP cells, which is normalized to the NTC samples transfected in parallel by flow cytometry analysis. Abbreviations: QC: Quinacrine; NS: Nucleostemin, IP/IB: immunoprecipitation/immunoblot; IgG: Immunoglobulin G control; DS: double-strand break; GFP: green fluorescent protein; NTC: non-targeted control, sh_NS: shRNA-mediated NS downregulation; OVCAR8-DR-GFP: OVCAR8 cells with stable integration of pDR-GFP plasmid; I-Sce: Intron-encoded endonuclease.

Based on the prior reports that NS recruits RAD51 to DNA damaged sites [24,25], we hypothesize that QC-induced downregulation of NS may compromise RAD51 recruitment and HR repair, thereby increasing the sensitivity. We found that NS and RAD51 interact in parental OVCAR5 and Tyk-nu cells (Figure 4A). This was further supported by NS and RAD51 co-localization in OVCAR5 cells (Figure 4B), but with the addition of QC decreased protein detection and RAD51 diffused in the nucleus (Figure 4C).

The NS interaction with RAD51 suggested that QC-induced downregulation of NS may compromise HR repair, and this was confirmed by the PCR-based HR assay [47]. Exogenous expression of pCβAI-Sce I plasmid in cells transfected into OVCAR-8DR-GFP cells will lose the Sce1 site if repaired due to HR and will appear as an uncleaved product by PCR. Following 72 h of QC treatment, DNA was extracted and the presence of the I-SceI cleaved product, which cleaves between non-functional GFP repeats, indicated inhibition of HR repair as visualized on agarose gels [54] (Figure 4D,E). To confirm that this HR inhibition could be though a reduction in NS, we transiently knocked down NS expression in OVCAR-8DR-GFP cells. Two days after transduction, I-Sce I plasmids were electroporated in these cells and analyzed for GFP fluorescence on day five (Figure 4F). The reduction in GFP positive cells in NS-knockdown cells indicates reduced HR-mediated repair of stably integrated HR substrate-DR-GFP (Figure 4G).

### 3.4. QC Treatment Downregulates NS Parylation and Induces DNA Damage in OC Cells

**Figure 5:**
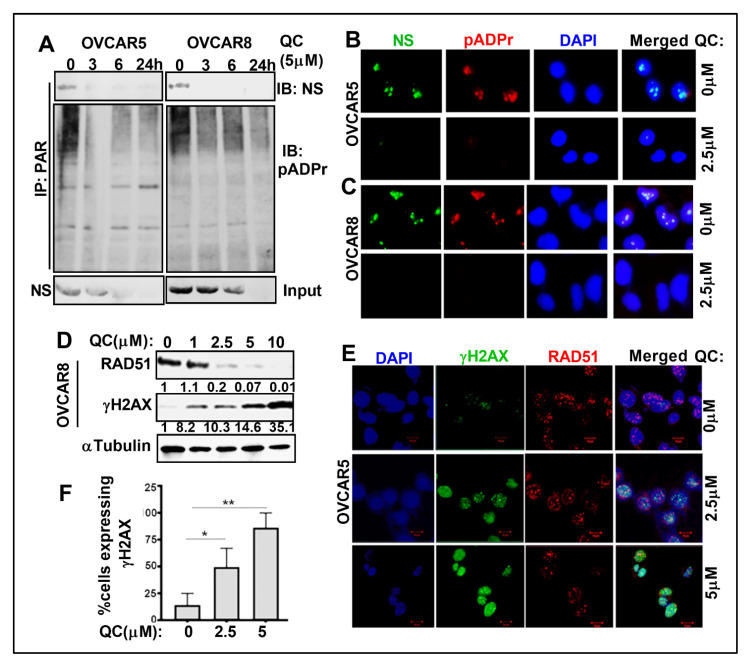
QC downregulates NS PARylation. and induces DNA damage. (**A**) OVCAR5 and 8 cell extracts were immunoprecipitated with anti-PAR antibody from untreated and QC-treated cells and probed for NS PARylation levels in a time dependent manner. (**B**) IF analysis shows NS (green) and pADPr (Red) colocalizes with each other (Merged image) and DAPI was used to stain the nuclei in OVCAR5 and (**C**) OVCAR8 cells. (**D**) Western analysis was performed in OVCAR8 cells to show the dose dependent increase in γH2AX levels and concomitant reduction in RAD51 levels upon QC treatment. αTubulin was used aa loading control. Densitometric analysis was performed using Image J, normalized and fold change was provided beneath the panel. (**E**) Representative IFC images of OVCAR5 cells untreated and treated with 2.5 and 5.0 µM QC showing γH2AX (green) and RAD51 (Red). DAPI-stained nuclei are in blue. (**F**) Quantitation of γH2AX levels. The number of cell nuclei displaying <5 foci (negative), between 6 and >20 foci, and diffuse pan-nuclear staining for pH2AX and RAD51 foci was quantified. At least 50 cells were counted (×40) for drug treatment per experiment. * (*p* < 0.05), ** (*p* < 0.01). Abbreviations: QC: Quinacrine; NS: Nucleostemin; IP/IB: immunoprecipitation/immunoblot; PAR: protein-conjugated polymers of ADP-ribose; pADPr: Poly(ADP-ribose) Polymer; H2AX: H2A histone family member X.

Upon DNA damage, PARP1 that is rapidly recruited to DNA damaged sites is autoPARylated when it induces the synthesis of protein-conjugated polymers of ADP-ribose (PAR). The PAR chains on PARP1 serve as a platform for the recruitment of downstream repair factors in the BER/SSB pathway. It is unknown if QC can modulate PAR levels, or if NS is PARylated and can recruit RAD51 to DNA nicks. Using the anti-PAR antibody, we have determined that PAR immunoprecipitates NS and the NS PARylation levels were completely abrogated upon 3 h QC treatment and was sustained through 24 h in OVCAR5 and OVCAR8 cells, with total NS levels downregulated by 6 h (Figure 5A). This was confirmed by QC-induced disruption of NS co-localization with PAR (Figure 5B,C). As a control experiment, rucaparib and olaparib PARPis were used to inhibit PARylation without a significant reduction in NS levels (Figure S5C,D). Based on these data, we surmise that PARylation. of NS may have a role in the recruitment of RAD51 to DNA damaged sites. QC-induced NS downregulation and inhibited PARylation may promote DNA damage in the cancer cells. In addition, we found that QC treatment promotes increased γH2AX and reduced RAD51 levels in OVCAR8 cells (Figure 5D and Figure S5E,F) and OVCAR5 cells (Figure S6A,B). Similar results were obtained for increased γH2AX foci formation by immunofluorescence assay upon QC treatment in OVCAR5 cells (Figure 5E,F).

### 3.5. NS Knockdown Cells Increased QC-Induced DNA Damage and QC Sensitizes Cells to PARPi

**Figure 6:**
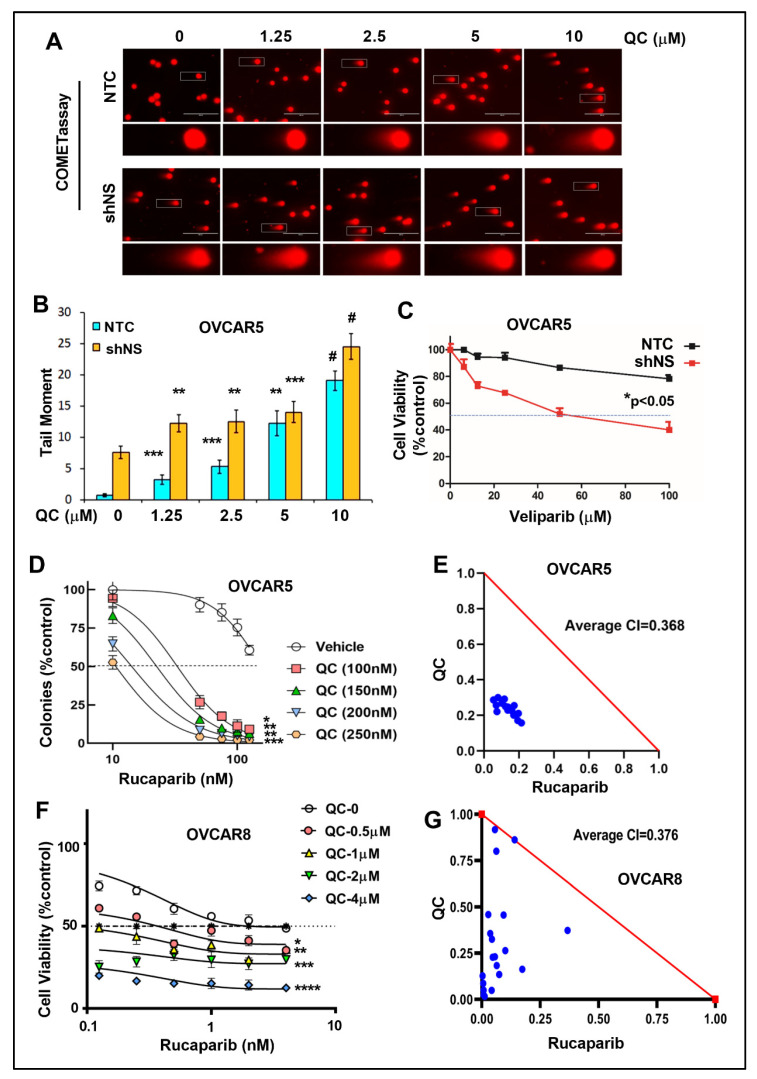
NS knockdown cells are sensitized to QC-induced DNA damage and QC sensitizes OC cells to PARPi. (**A**) Alkaline COMET assay shows increase in DSDBs following QC treatment in both OVCAR5 NTC and NS KD cells. (**B**) Quantified tail moment from the COMET assay was represented ** (*p* < 0.001), *** (*p* < 0.0001), ^#^ (*p* < 0.00001). (**C**) Cell viability was assessed in both NTC and NS KD cells upon dose dependent Veliparib treatment using MTT assay. (**D**) Colony formation assays using OVCAR5 cells upon treatment with indicated concentrations of QC alone or in combination with Rucaparib was shown. * (*p* < 0.01), ** (*p* < 0.001), *** (*p* < 0.0001). (**E**) The combination index (CI) was plotted as a function of dose combination, with average CIs for the drug combination reported in panel. The additive isobole is depicted in this panel as a red straight line, with synergistic dose combinations labeled below the isobole. An average CI of 1 indicates an additive effect, CI < 1 a synergistic effect, and CI > 1 an antagonistic effect. (**F**) Cell viability assay for 48 h using OVCAR8 cells upon treatment with indicated concentrations of QC alone or in combination with Rucaparib was shown. * (*p* < 0.01), ** (*p* < 0.001), *** (*p* < 0.0001), **** (*p* < 0.00001). (**G**) The combination index (CI) was plotted as mentioned above. Abbreviations: QC: Quinacrine; NS: Nucleostemin; NTC: non-targeted control, sh_NS: shRNA-mediated NS downregulation; COMET: single cell gel electrophoresis assay.

The effect of NS knockdown in QC-induced DNA damage was assessed by the alkaline COMET assay. QC treatment induced more DNA damage in OVCAR5 NS knockdown cells compared to the NTC controls (Figure 6A,B). Additionally, we examined whether knockdown of RPA194 also affected the sensitivity of OC cells to QC treatment. In OVCAR7 cells with RPA194 shRNA (Figure S6C), the colony forming ability was assessed to measure the sensitivity for both the OVCAR7 RPA194 knockdown clones and the NTC control cells upon treatment with increasing concentrations of QC (Figure S6D). Increased inhibition of cell viability was observed in the RPA194 knockdown cells compared to NTC controls with a shift in QC IC50 values to a low range in the knockdown cells (Figure S6E).

We evaluated the cell death response when PARPi was combined with NS knockdown or QC combination. The NS knockdown OVCAR5 cells were more sensitive to increasing concentrations of veliparib compared to the NTC control cells (Figure 6C). To determine if QC synergizes with PARPi, we analyzed the effects on OVCAR5 cells upon treatment with indicated concentrations of QC alone or in combination with rucaparib using colony formation assays. The combination of QC (0–250 nM) with rucaparib (0–125 nM) was strongly synergistic with average CI at 0.36 in OVCAR5 cells (Figure 6D,E). Cell viability assay by MTT was performed in OVCAR8 cells with the combination of QC (0–4000 nM) with rucaparib (0–4000 nM) and was found to be highly synergistic with average CI at 0.37 (Figure 6F,G). Since OVAR5 and 8 cell lines are considered resistant to PARPis with no reported alterations in any of the DNA repair genes including BRCA1 and 2 (Cancer Cell Line Encyclopedia (BROAD, 2019) on the cbioportal.org), we used these cell lines for the PARPi synergy study with QC. Together, these results provide evidence that QC, by downregulating RBG and inducing nucleolar stress, increases DNA damage and sensitizes OC cells to PARPis.

## 4. Discussion

Chemotherapy resistance in OC and the drug-refractory nature of high-grade serous OC are major obstacles for OC patient survival. We have previously reported that QC promotes autophagic cell death and chemosensitivity in OC [31], which led to this RNA-seq comparison for isogenic resistant cell lines upon QC treatment. RNA-seq analysis and the validation of selected genes suggest QC to regulate RBG. A prior report on QC regulation of the RNA Pol I subunits in acute myeloid leukemia cells compliments our findings [32]. This is the first report, to our knowledge, of QC-induced regulation of RBG in solid tumors, as well as with an emphasis on nucleolar stress in therapy-refractory cancer cells. To highlight the preclinical efficacy of QC in resistant-ovarian cancer treatment our previously published study has already shown the sensitization of the three resistant cell types to the drugs they are resistant to, upon treatment with QC [31]. Constant ratio synergy studies performed in isogenic cisplatin sensitive OV2008 and cisplatin resistant C13 cells by treating them with a combination of cisplatin QC, showed a more potent synergistic antiproliferative effect in C13 compared to OV2008 cells (refer to Figure 4A,B in [31]). Similar studies with isogenic taxol-sensitive SKOV3 and taxol-resistant SKOV3TR cells, also indicate that QC has a more synergistic antiproliferative effect when combined with either cisplatin or carboplatin in SKOV3TR compared to SKOV3 cells (refer to Figure 4C,D in [31]). Carboplatin-resistant HeyA8MDR cells also showed stronger synergy when carboplatin was combined with QC compared to the parent chemosensitive HeyA8 cells (refer to Figure 4E,F in [26]). We have also shown that QC synergizes with carboplatin to reduce tumor burden in the HeyA8MDR-derived mouse xenograft model (refer to Figure 7 in [31]).

Selected QC-induced expression changes, including BOP1, were validated and QC modulation of RBG was confirmed by measuring RNA subunits and 5-fluorouracil incorporation. The QC-induced redistribution of FBL and downregulation of NS suggested nucleolar stress conditions in high-grade serous OC cells. Nucleolar stress is an emerging concept where nucleolar functions may sense various cellular stresses that impair RBG and activate stress-responsive signaling [52]. RBG can be suppressed at RNA Pol I initiation, pre-ribosomal RNA processing, and ribosomal assembly stages, which can also be impacted by many other changes such as physiochemical stressors and autophagy dysregulation (e.g., key autophagic protein LC3 localizes to nucleolus). The canonical output of nucleolar stress is p53 signaling activation. Our interim hypothesis that QC induces nucleolar stress was based on its modulation of RBG [32], autophagy [31], and p53 activation [27]. However, TP53 is mutated in about half of all cancer types and >95% of high-grade serous OC tumors [55]. As QC is cytotoxic in TP53-null OC cells36, we identified NS as its alternative downstream target. NS is a nucleolar protein that regulates both p53 signaling and pre-ribosomal RNA processing [53].

The RNA Pol I-induced transcription of ribosome production in the nucleolus is frequently upregulated in cancer cells, which supports the theory that cancer cells are addicted to this process to accommodate for the increasing demands on protein synthesis, growth, and proliferation. This may open a therapeutic window to specifically target cancer cells with minimal effect to normal cells. The loss of tumor suppressors (e.g., p53) can result in hyperactivation of RBG, and its inhibition leads to nucleolar stress response to promote p53 stabilization, cell cycle arrest, and apoptosis. All these processes, when normal regulation is lost in cancer cells, culminate in phenotypic hallmarks of cancer.

Previous studies have demonstrated that QC inhibits RBG in leukemia cells, although it is unclear whether it has similar effect in solid tumors [32,34]. QC targets several signaling pathways simultaneously by affecting autophagy, apoptosis, p53, AKT, NFκB, HSF1, and methylation pathways, all of which are pathways implicated in OC chemoresistance [29]. QC reduces the multidrug resistance phenotype in the ovary, prostate, and leukemic cells [56]. In total, QC shows anticancer activity in cervical [57], colon [58], glioma [59], breast [60], ovarian [61], and leukemia [32] cancers. We have previously shown QC inhibits OC growth in vivo [31] and in vitro by modulating cell cycle proteins p21 and Skp2 [43] in an autophagy-dependent and p53-independent manner. In this study, we demonstrate that QC also targets treatment-refractory OC cells by inhibiting RBG. A recent report by Eriksson et al. showed that QC-induced gene expression shows the highest correlation to that of ellipticine, an RNA Pol I inhibitor [32,33]. Considering that Pol I is essential for RBG, we explored the extent to which QC inhibits RBG and produces therapeutic effects through nucleolar stress. Nucleolar size, irregularity, and number are determinants in tumor grade and are associated with aggressive tumor behavior and poor outcome, underscoring the significance of nucleolar function in tumorigenesis. The nucleolus, the hub for RBG, is also the site for RNA Pol I-driven rDNA transcription. Inhibition of Pol I-driven transcription of ribosomal genes induces nucleolar stress and results in the disruption of nucleolar structures [62]. This leads to the translocation of resident nucleolar proteins, including NS and FBL as well as several ribosomal proteins, into the nucleoplasm where they bind MDM2 to induce p53 stabilization, cell cycle arrest, and apoptosis. Several chemotherapeutic drugs concomitantly inhibit RBG to induce nucleolar stress [12] and induce DNA damage [13]. The nucleolus is increasingly being recognized as playing an active role in the DNA damage response due to the localization of proteins involved in DNA repair including PARP1, BRCA1, NS, and 5′-flap endonuclease and 3′-5′ exonuclease (FEN1) [23]. rDNA repeats are particularly vulnerable to genomic instability, and a high rate of transcription at these loci due to increased RBG further increases the instability. Accumulating data suggest that NS, a stem cell-enriched nucleolar protein, promotes genome stability and protects against replication-induced DNA damage. Mechanistically, it was found to regulate the HR repair by recruiting RAD51 to DNA damage and, thus, conferring resistance to PARP inhibitors [24,25]. Herein, we found that QC downregulates NS, which inhibits RBG and DNA repair. QC-induced downregulation of NS disrupted HR repair by decreasing the NS protein levels and PARylation that resulted in reduced RAD51 recruitment to DNA damage. Overall, our data suggest that QC inhibits RBG and that this inhibition may promote DNA damage by downregulating NS. We also observed that QC strongly synergizes with PARPi in the ovarian cancer cells, which supports the development of this combination for future OC treatment.

## 5. Conclusions

In conclusion, we report that QC-induced nucleolar stress and inhibition of RBG resulted in reduced OC cell proliferation and DNA damage. Our RNA-seq analyses, followed by a further pathway investigation, demonstrate that QC induces nucleolar stress in therapy-refractory OC models. Understanding the anticancer mechanisms of QC will support clinical trials and optimal indications for repurposing this drug, and the potential of QC to be combined with additional drugs such as PARP inhibitors for the benefit of OC patients.

## Data Availability

RNA-seq raw data files were submitted in the NCBI Gene Expression Omnibus (GEO) and accessible under GEO series accession number GSE176246.

## Supplementary Materials

The following are available online at https://www.mdpi.com/article/10.3390/cancers13184645/s1. Table S1: Cell Culture Details Provided, Table S2: Source of Antibodies and Reagents Used, Table S3: Primer Sequence Table. Figure S1: Differential gene expression in chemotherapy sensitive and resistant isogenic cells by QC treatment. Figure S2. Validation of expression for selected genes in the RBG pathway with and without QC treatment. Figure S3: QC induces nucleolar stress. Figure S4: QC and CX5461 ribosomal inhibitor downregulates NS. Figure S5: PARPis downregulates parylation of NS. Figure S6. RPA194 knockdown cells are sensitized to QC treatment.
