# Supplementary material for: Quinacrine Induces Nucleolar Stress in Treatment-Refractory Ovarian Cancer Cell Lines
Source: Cancers (Basel). 2021 Sep 16;13(18):4645. doi: 10.3390/cancers13184645 (PMC8466834; doi:10.3390/cancers13184645)
Supplement: Supplementary file 1 [file cancers-13-04645-s001.zip › cancers-1312833-supplementary.pdf]

Quinacrine induces nucleolar stress in treatment-refractory ovarian cancer cell lines

Derek B. Oien [1,†], Upasana Ray [1], Christopher L. Pathoulas [1,2], Ling Jin [1], Prabhu Thirusangu [1], Deokbeom Jung [1,3], Joseph E. Kumka [1,4], Yinan Xiao [1,5], Sayantani Sarkar Bhattacharya [1], Dennis Montoya [6], Jeremy Chien [6] and Viji Shridhar [1,*]

1 Division of Experimental Pathology and Laboratory Medicine, Mayo Clinic, 200 First Street SW, Rochester, MN 55905, USA; derek.oien@astrazeneca.com (D.B.O.); ray.upasana@mayo.edu (U.R.); pathoulas@uchc.edu (C.L.P.); jin.ling1@mayo.edu (L.J.); Thirusangu.Prabhu@mayo.edu (P.T.); jungdb@hotmail.com (D.J.); Joseph.kumka@gmail.com (J.E.K.); yinanxiao1203@gmail.com (Y.X.); bhattacharya.sayantani@mayo.edu (S.S.B.)

2 University of Connecticut Health Center-Medical School, Farmington, CT 06032, USA

3 ASAN Biomedical Research Center, Seoul 138-736, Korea

4 University of Minnesota Medical School, Minneapolis, MN 55455, USA

5 Department of Obstetrics and Gynecology, The Second Xiangya Hospital, Central South University, Changsha 410008, China

6 Department of Biochemistry and Molecular Medicine, University of California Davis Health, 2700 Stockton Boulevard, Sacramento, CA 95817, USA; djmontoya@ucdavis.edu (D.M.); jrchien@ucdavis.edu (J.C.)

* Correspondence: shridhar.vijayalakshmi@mayo.edu

† Current address: Oncology R&D, AstraZeneca, Boston, MA 02451, USA

Table S1: Cell culture conditions.

Cell lines	Media	Supplements
OVCAR 4/5/7/8	RPMI-1640	10% FBS and 1% Pen/Strep
PEO1/PEO4	RPMI-1640	10% FBS and 1% Pen/Strep
OV2008/C13	RPMI-1640	10% FBS and 1% Pen/Strep
SK-OV-3/ SK-OV-3-TR	RPMI-1640	10% FBS and 1% Pen/Strep
HeyA8/HeyA8MDR	DMEM (4.5 g/l glucose)	10% FBS and 1% Pen/Strep

Table S2: Details of antibodies and reagents used.

Antibodies	Company	Catalog No.
1. RPA194	Santa Cruz, Dallas, TX	sc-46699
2. NS	Santa Cruz Biotechnology, Texas, U.S.A	sc-166460
3. PCNA	Santa Cruz Biotechnology, Texas, U.S.A	sc-9857
4. RAD51	Abcam, Cambridge, UK	ab133534
5. H2AX	Santa Cruz Biotechnology, Texas, U.S.A	sc-517336
6. α-tubulin	Santa Cruz Biotechnology, Texas, U.S.A	sc-5286
7. GAPDH	Santa Cruz Biotechnology, Texas, U.S.A	sc-47724
8. BrdU	Bioss, Woburn, MA	BS-0489R
9. Fibrillarin	Santa Cruz Biotechnology, Texas, U.S.A	sc-166001
10. DNMT3B	LifeSpan Biosciences, Inc., WA, U.S.A	LS-C523
11. ASNS	Santa Cruz Biotechnology, Texas, U.S.A	sc-365809
12. CSTM	Santa Cruz Biotechnology, Texas, U.S.A	sc-73881
**Reagents**	**Company**	**Catalog No.**
1. 5-fluorouridine	Sigma-Aldrich	F5130
2. Quinacrine (QC)	Sigma-Aldrich	Q3251
3. TRIzol	Ambion, Carlsbad, CA	15596018
4. Chloroform	Sigma-Aldrich	C2432
5. Puromycin	EMD Millipore, Burlington, MA	A1113803
6. 3-(4,5-dimethylthiazol-2-yl)-2,5-diphenyltetrazolium bromide (MTT)	ThermoFisher Scientific	M6494
7. Antifade mounting medium with DAPI	Vectashield, Burlingame, CA USA	H-1200-10
8. QuantiTect Reverse Transcription cDNA Synthesis kit	Qiagen, Germantown, MD	#205311
9. iTaq Universal SYBR Green Supermix	Bio-rad, CA	#1725121
10. Fetal Bovine Serum (FBS)	Biowest	#S181A
11. 100µg/ml streptomycin and 100U/ml penicillin	Thermo Fisher Scientific	15070063

12. DMEM (4.5 g/l glucose),<br>13. RPMI-1640	Thermo Fisher Scientific	#11965118,<br>#11875093
14. Lipofectamine 3000	Thermo Fisher Scientific	L3000015

Table S3: Primer sequence for QPCR.

Gene	Forward Primer	Reverse Primer
GLN3	5'-AGC AGA AAC TTG ACA GGC AG -3'	5'-GAA TTC TGT TTG CCC GAC TTG-3'
ASNS	5'-GGA GAG TGA GAG GCT TCT G-3'	5' GGT GGC AGA GAC AAG TAA TAG G-3'
BOP1	5'-CAG TTC CTG GAC AAG ATG GAC-3'	5'-GTT GAA GCC CAC ATC CCC-3'
CTSM	5'-GGC AGC AAC AGC ATC TAC TAC-3'	5'-CAT CTC CAT CGT CAG GAA GTA C-3'
DNMT3B	5'-CCC ATT CGA GTC CTG TCA TTG-3'	5'-TTG ATA TTC CCC TCG TGC TTC-3'
PHDGH	5'-CAT CAG TGA CAG CCT GGA C-3'	5'-TGG CAG AGC GAA CAA TAA CG-3'
18s rRNA	5'-AAA CGG CTA CCA CAT CCA AG-3'	5'-CCT CCA ATG GAT CCT CGT TA-3'
28s rRNA	5'-TGG GTT TTA AGC AGG AGG TG-3'	5'-AAC CTG TCT CAC GAC GGT CT-3'
5'ETS	5'-GAA CGG TGG TGT GTC GTT-3'	5'-GCG TCT CGT CTC GTC TCA CT-3'
RPLP0	5'-ACC TCC TTT TTC CAG GCT TT-3'	5'-CCC ACT TTG TCT CCA GTC TTG-3'

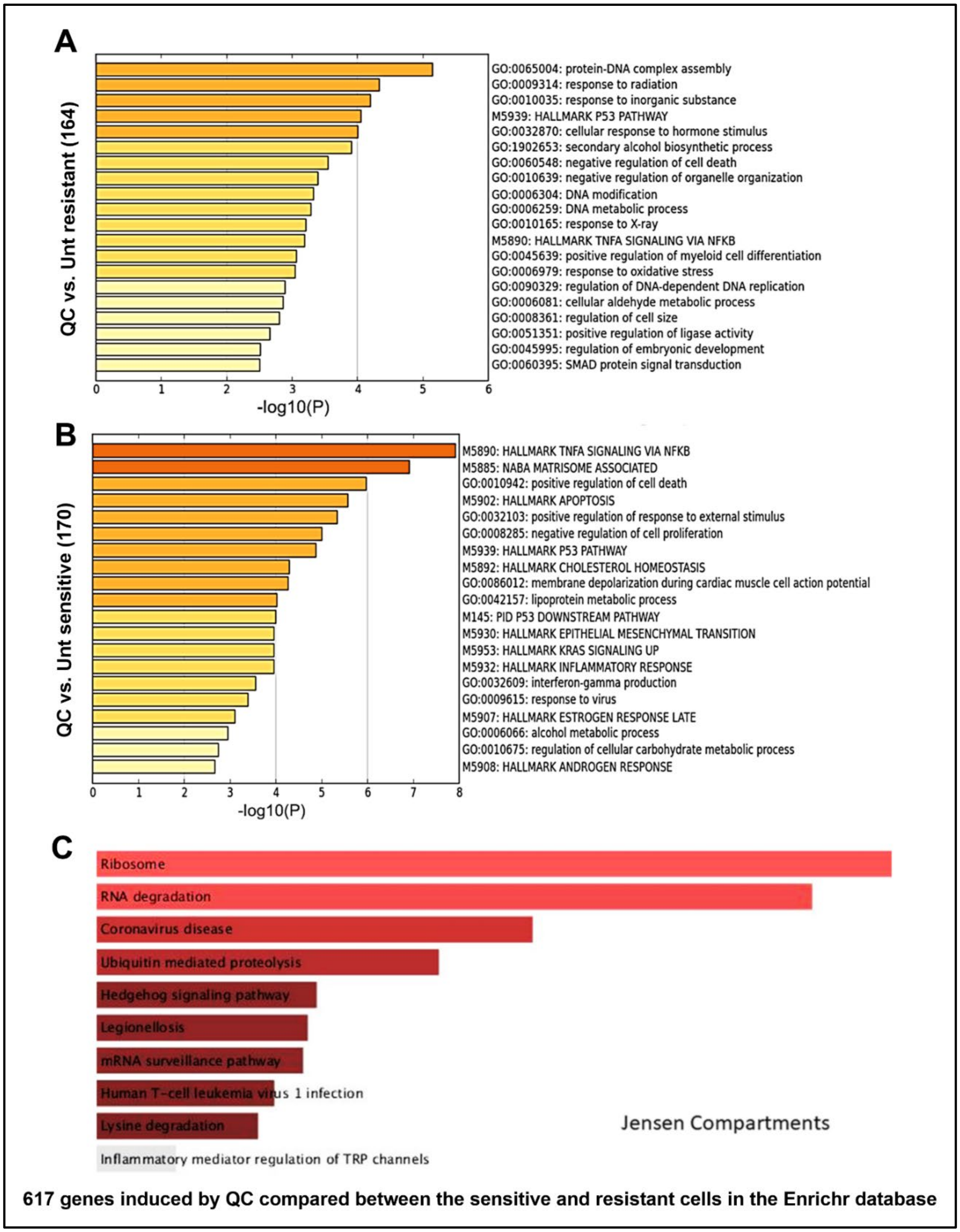

Figure S1: Differential gene expression in chemotherapy sensitive and resistant isogenic cells by QC treatment. (A) Pathway analyses for differentially expressed genes after quinacrine treatment of resistant cells and (B) sensitive cells. Graphs are output data of Metascape software (metascape.org) that show p-

value (as -log10(P)) for top associated pathways by significance to expression correlation. (C) 617 genes induced by QC that differed between the sensitive and resistant cells were analyzed in the Enrichr database and the pathway analysis presented as Jensen Compartments.

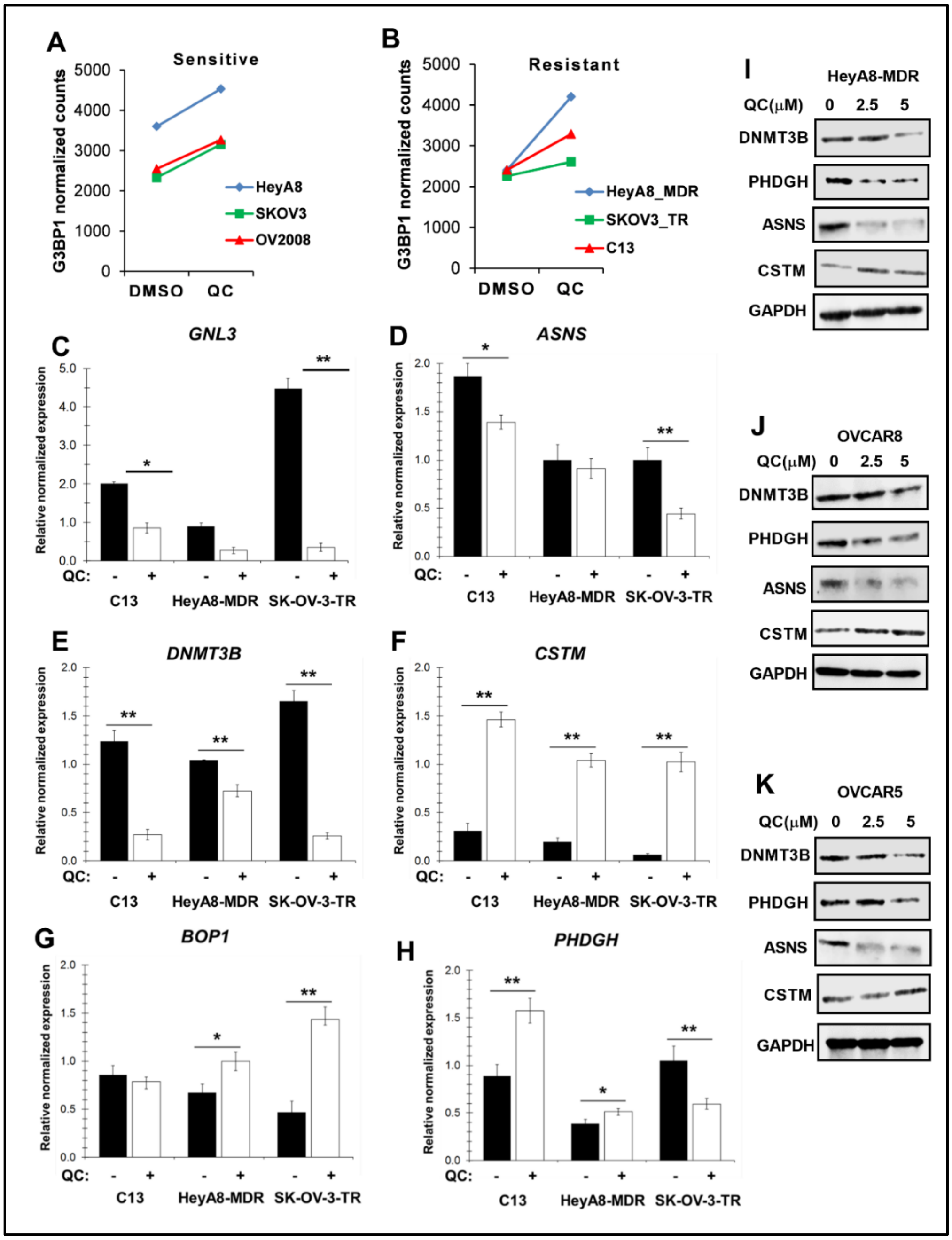

Figure S2: Validation of expression for selected genes in the RBG pathway with and without QC treatment. Genes expression analysis from RNA Seq data showed that G3BP1 is increased in sensitive

and resistant cells treated with quinacrine compared to control. Quantitative real-time PCR detecting mRNA expression in sensitive and resistant OC cells against (C) GNL3, (D) ASNS, (E) DNMT3B, (F) CSTM, (G) BOP1 and (H) PHDGH. Error bars represent standard error (n = 3), and significance is denoted by * ($p<0.05$) or ** ($p<0.005$). Western blot analysis for DNMT3B, PHDGH, ASNS and CSTM protein level changes upon QC treatment was analyzed in (I) HeyA8-MDR, (J) OVCAR8 and (K) OVCAR5 cells. GAPDH was used as loading control.

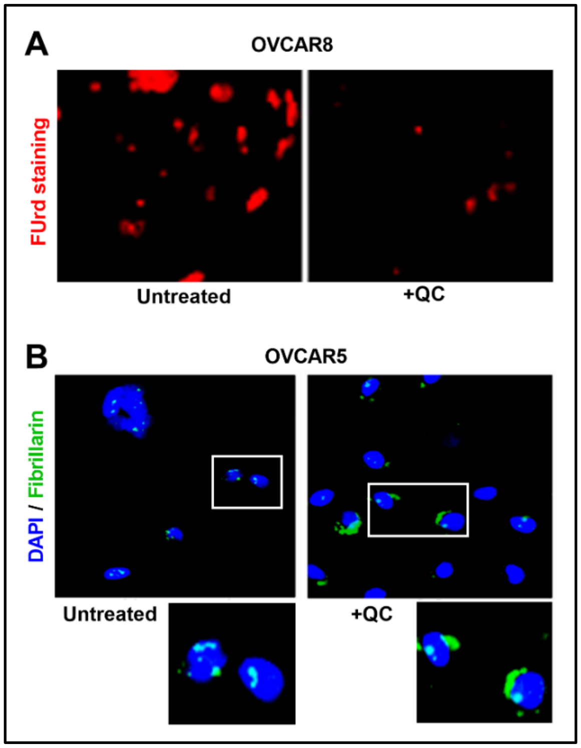

Figure S3: QC induces nucleolar stress. (A) Incorporation of 5-fluorouridine was monitored after 10minutes of labeling in OVCAR8 cells treated with 1µM QC for 2hrs. Representative images were shown. (B) Fibrillarin (Green) IF staining was performed after 2hrs of 1µM QC in OVCAR5 cells. DAPI was used to stain nucleus.

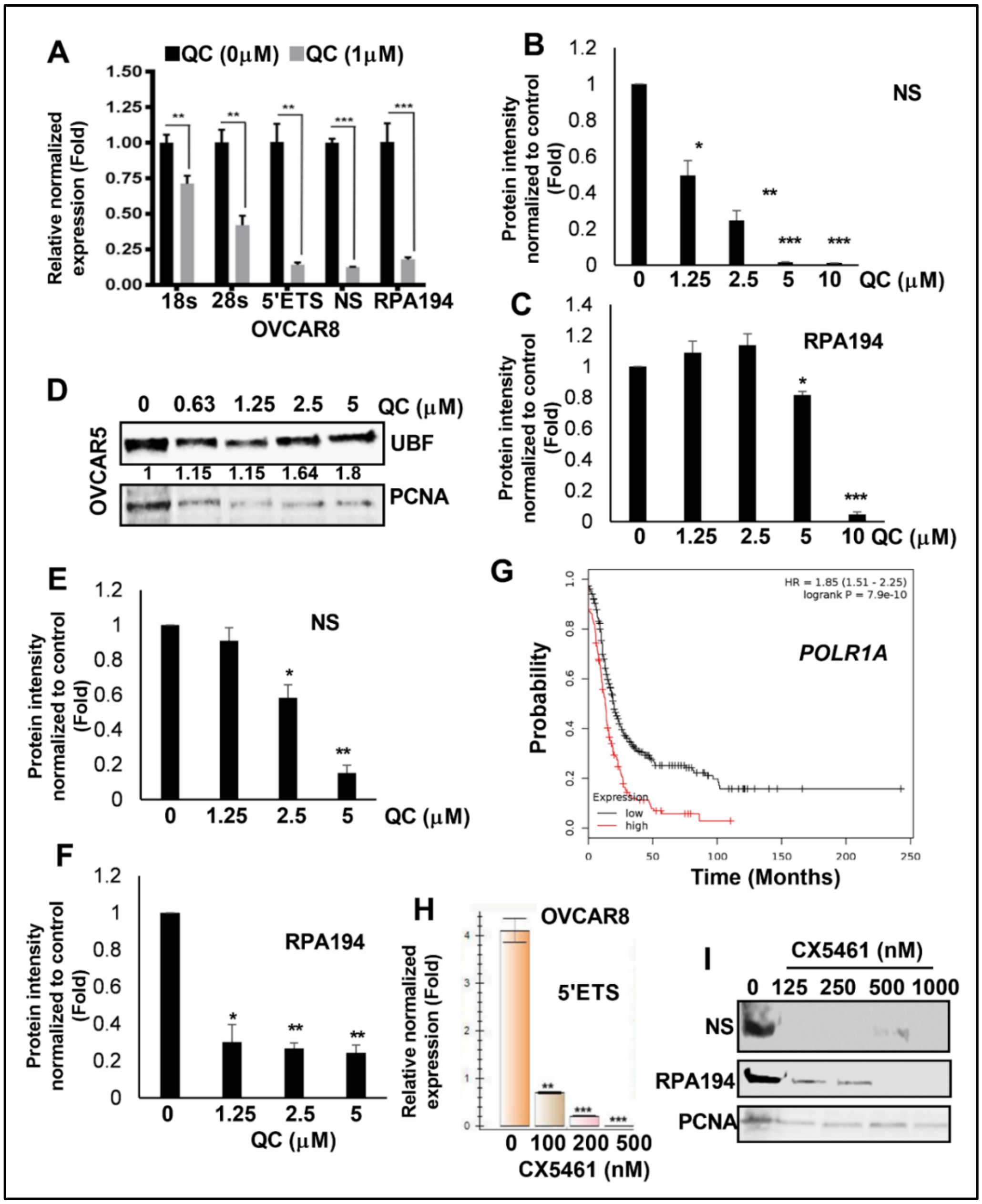

Figure S4: QC and CX5461 ribosomal inhibitor downregulates NS. (A) Relative expression changes for ribosomal subunits, NS and RPA194 after 1hr of 1µM QC treatment in OVCAR8 OC cells (normalized to RPLP0; n=3) *(p<0.05), ** (p<0.01), ***(p<0.001). (B-C) Graphs show the densitometric analysis normalized to endogenous control for fig.3D. (*p < 0.05 **p < 0.01 ***p < 0.001). (D) Western blot analysis for UBF upon treatment with indicated dose of QC for 24hrs. PCNA was used as loading control. (E-F) Densitometric plots normalized to endogenous control for fig.3E. (*p < 0.05, **p < 0.01).

(G) Kaplan-Meier progression free survival (PFS) analysis shows high POLR1A expression is associated with worse PFS in OC patient's cohort. (H) Relative expression changes for 5'ETS after 24hr of increasing concentration of CX5461 treatment in OVCAR8 cells (normalized to RPLP0; n=3). (I) Immunoblot showing CX5461 treatment downregulates NS and RPA194 expression in OVCAR5 cells in a dose dependent manner. PCNA was used as an endogenous control.

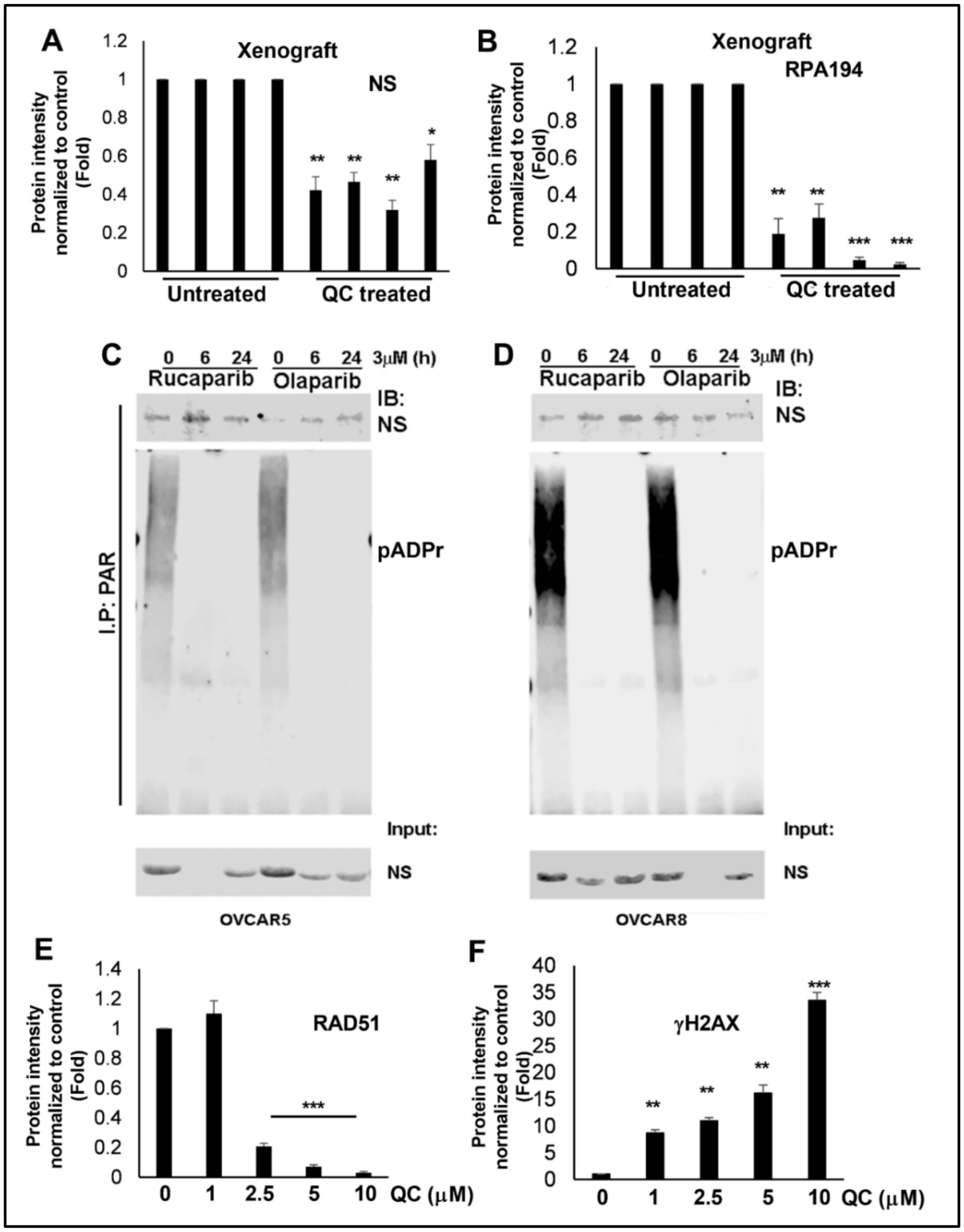

Figure S5: PARPis downregulates parylation of NS. (A-B) Graphs show the densitometric analysis normalized to endogenous control for fig.3I (*p < 0.05 **p < 0.01 ***p < 0.001). (C) OVCAR5 and (D)

OVCAR8 cell extracts were immunoprecipitated with anti-PAR antibody from untreated and Rucaparib/Olaparib treated cells and probed for NS and NS parylation levels pADPr in a time dependent manner. (E-F) Densitometric plots normalized to endogenous control for fig.5D. (**p < 0.01 ***p<0.001).

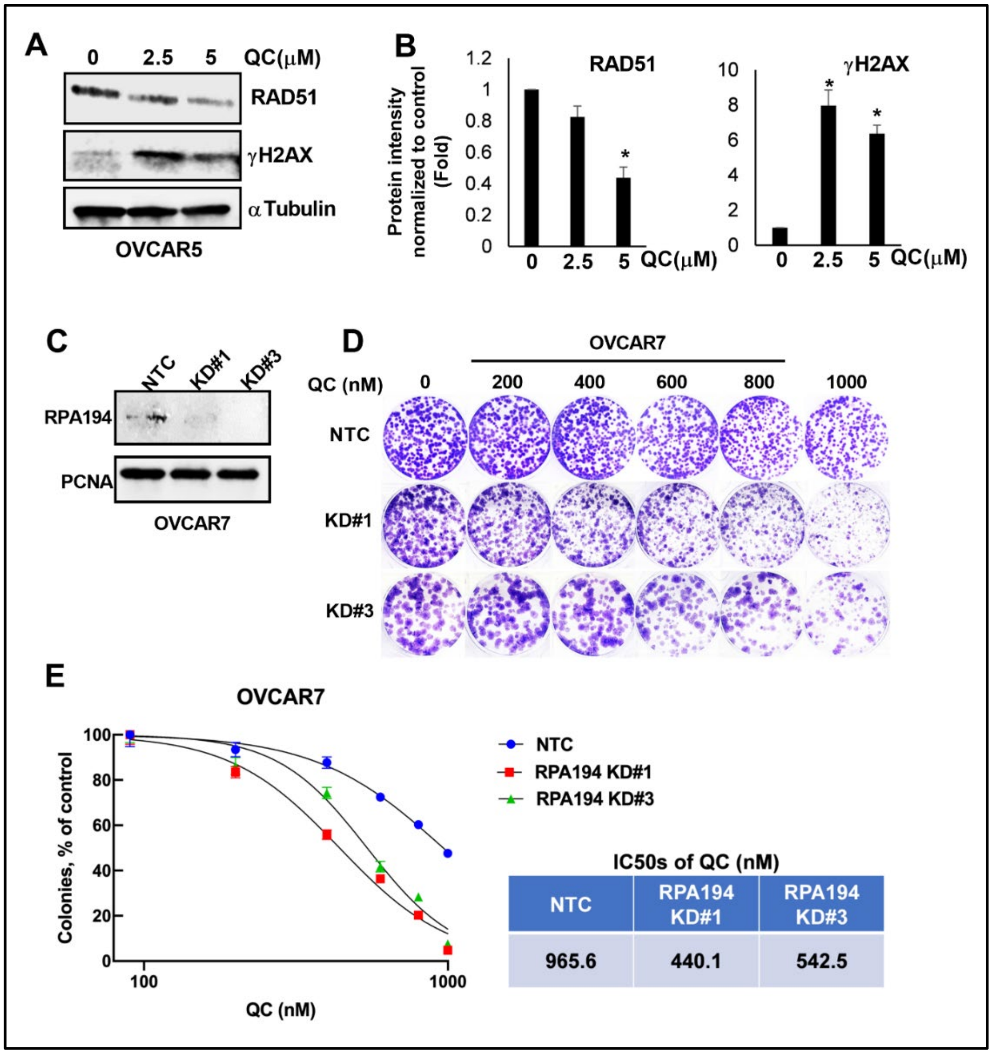

NTC	RPA194 KD#1	RPA194 KD#3
965.6	440.1	542.5

Figure S6: RPA194 knockdown cells are sensitized to QC treatment. (A) Western analysis was performed in OVCAR5 cells to show the dose dependent increase in γH2AX levels and concomitant reduction in RAD51 levels upon QC treatment. αTubulin was used aa loading control. (B) Densitometric analysis was performed using Image J, normalized and fold change was plotted (*p < 0.05). (C) RPA194 shRNA mediated stable knockdown OVCAR7 cells were generated and the effective knockdown was

validated by immunoblot analysis. (D) Clonogenic survival assays with or without QC treatment was performed in NTC control and shRPA194 OVCAR7 cells. Images captured and shown. (E) Colonies counted, data was analyzed and plotted and the IC50 value was determined in all the treatment sets.
